# Diffusion tensor imaging of white-matter structural features of maltreating mothers and their associations with intergenerational chain of childhood abuse

**DOI:** 10.1038/s41598-024-53666-0

**Published:** 2024-03-07

**Authors:** Sawa Kurata, Shota Nishitani, Natasha Y. S. Kawata, Akiko Yao, Takashi X. Fujisawa, Hidehiko Okazawa, Akemi Tomoda

**Affiliations:** 1 Division of Developmental Higher Brain Functions, United Graduate School of Child Development, Osaka University, Osaka, Japan; 2 Kanazawa University, Kanazawa, Japan; 3 Hamamatsu University School of Medicine, Hamamatsu, Japan; 4 Chiba University, Chiba, Japan; 5 University of Fukui, Osaka, Japan; 6 Department of Child and Adolescent Psychological Medicine, University of Fukui Hospital, Fukui, Japan; 7 Research Center for Child Mental Development, University of Fukui, Fukui, Japan; 8 Life Science Innovation Center, School of Medical Sciences, University of Fukui, Fukui, Japan; 9 Biomedical Imaging Research Center, University of Fukui, Fukui, Japan

**Keywords:** Neuroscience, Psychology

## Abstract

Child abuse causes lifelong adverse outcomes for both physical and mental health, although many are resilient. Efforts to prevent this issue from the parental side require an understanding of the neurobiological basis that leads abusive parents to perpetrate abuse and the influence of the intergenerational chain of childhood abuse. Therefore, this study was conducted to compare the brain white-matter fiber structures between 11 maltreating mothers who had been recognized as having conducted child abuse prior to the intervention and 40 age-matched control mothers using tract-based spatial statistics. There was a significantly reduced axial diffusivity (AD) and a similar trend in fractional anisotropy (FA) in the right corticospinal tract in maltreating mothers compared to control mothers. Therefore, maltreating mothers may have excessive control over the forcefulness of voluntary movements. These features also decreased as the number of childhood abuse experiences increased, suggesting that an intergenerational chain of child abuse may also be involved. Other aspects observed were that the higher the current depressive symptoms, the lower the AD and FA values; however, they were not related to parental practice or empathy. These results corroborate the neurobiological features that perpetrate behaviors in abusive mothers.

## Introduction

Child maltreatment is an essential global social issue affecting public health and social welfare. Up to one billion (12.6%) children aged 2–17 years have been victims of physical, emotional, or sexual abuse or neglect worldwide in the past year^1^. The accumulation of their traumatic experiences has been reported to be concerning, with many reports of lasting impact on the development of their brain structure and function, although many are resilient, resulting in severe and long-term adverse effects on psychosocial development^2^. Therefore, studies in a variety of fields, from epidemiology to neurobiology, have been attempting to identify risk factors underlying perpetrating child maltreatment^3,4^.

Epidemiological studies have revealed parental economic deprivation, marital status, number of children, young age, low educational level, substance abuse, and caregiver mental illness as risks for child maltreatment^5^. However, they are not necessarily directly linked to maltreatment, which led to various neurobiological studies that go beyond this to identify biological risk factors. Among them, brain imaging studies on mothers have been explored primarily because of a growing interest in psychological topics, including brain structures that specifically change when women become mothers and brain functions that reflect feelings of love for their children^6–8^. However, few brain imaging studies have been conducted on maltreating mothers. There are several reasons for this: there is no diagnostic category for perpetrating child maltreatment in the Diagnostic and Statistical Manual of Mental Disorders (DSM)-5, which many psychiatric studies refer to; some parents may have various mental and personality disorders in their background; and the DSM-5 categories would make them a heterogeneous group that straddles many disorders; it is not easy to obtain informed consent for research participation, which may be why they have tended to be avoided.

However, studies have explored brain vulnerability in populations with high-risk factors epidemiologically associated with child maltreatment^9,10^. Connectivity between the posterior cingulate cortex and the amygdala is significantly reduced in mothers with postpartum depression compared to healthy mothers with low-frequency neural activity. It is associated with reduced empathic behavior and emotion regulation during mother-infant interactions^11^. Mothers with postpartum depression showed increased cortical thickness in the left superior frontal gyrus, cuneus, right lingual gyrus, and spindle gyrus compared with healthy postpartum women. It has also been suggested that focal swelling of the right globus pallidus is associated with negative parenting behaviors that cause apathy and indifference^12^. Reports on postpartum depression and major depression indicate brain vulnerabilities that can be risk factors for child maltreatment^13^. However, these findings do not necessarily directly lead to maltreatment. Besides, multiple reports state that borderline personality disorder (BPD) is associated with an increased tendency to be a child maltreatment perpetrator due to its difficulty in controlling emotions^14–17^. It has been suggested that BPD shows reduced integration of some white matter fiber structures in the frontal limbic system, elucidating BPD-specific symptoms such as emotion dysregulation and cognitive dysfunction^18^. However, difficulty with emotion regulation does not necessarily directly lead to maltreatment. Therefore, brain imaging studies of maltreating mothers, rather than indirect studies, would still be necessary to understand what part of the core neuropathology leads to the perpetration of child maltreatment.

Based on the idea of the Research Domain Criteria (RDoC), which are not limited to traditional disease categories, a small number of case–control studies of domain groups defined by the social factors of maltreatment have been conducted. Brain structural Magnetic Resonance Imaging (MRI) studies of neglectful mothers have shown reduced gray matter volume (GMV) in the right insula, anterior/mid cingulate gyrus, and right inferior frontal gyrus, as well as reduced white-matter volume in the bilateral frontal regions by the voxel-based morphometry used T1 images when compared with a control group^19^. They also showed reduced activity in the right middle frontal gyrus and right cingulate gyrus in response to an infant's crying face stimulus presentation and blunted responses to the infant's emotions^20^. Neglectful mothers also possess reduced cortical thickness in the right middle frontal gyrus and orbitofrontal cortex, responsible for cognitive control and emotional processing^21^. These findings suggest that mothers with neglect perpetration may be vulnerable in frontal lobe regions associated with reduced functional connectivity in emotion-empathy-related areas and mother–child interactions^19^. To the best of our knowledge, other studies on maltreating mothers, particularly those examining white-matter fiber structures using diffusion-weighted imaging, are lacking. Diffusion MRI can infer features of the brain microstructure, such as microstructural complexity, integration, axonal density and variability, and degree of crystallization, via the displacement of water molecules diffusing within the tissue^22^. The diffusion of water molecules can be quantified by diffusion anisotropy (fractional anisotropy [FA]), which is the ratio of diffusion parallel to the fiber (axial diffusivity [AD]) to that perpendicular to the fiber (radial diffusivity [RD]). Therefore, we supposed that maltreating mothers have some differences compared to a control group in white-matter fiber structures. We primarily aimed to examine whole-brain exploratory analysis using diffusion-weighted imaging.

In contrast, a meta-analysis of epidemiological risk factors for perpetrating parents identified childhood abuse experiences by their parents, in addition to current problems of physical and mental health and socioeconomic status^23^. This is well known as an intergenerational chain of childhood abuse^24^. Therefore, in addition to the current prevalence of mental illness and the fact that abuse is committed, research has also been conducted to examine the brain structure and function related to the parents’ history of childhood abuse. Mothers who have experienced childhood abuse have been reported to be less sensitive in interaction with their children^25^. However, epidemiological statistics^24,26^ are inconsistent, with approximately 7–70% of maltreating parents having experienced childhood abuse. It is difficult to disentangle whether maltreatment perpetrates from an intergenerational chain of abuse or without adverse childhood experiences. However, if case–control analysis reveals differences in white matter fiber structure in maltreating mothers, examining whether those findings are related to a history of childhood abuse may allow us to interpret these features not only as a vulnerable neural basis for perpetration at present but also owing to their past childhood abuse experiences.

Therefore, as the primary aim, we compared the brain white-matter fiber structure in a case–control design between maltreating mothers (mothers defined as the child abuse perpetrator domain) and mothers in the general population to determine the presence or absence of features found in maltreating mothers. Secondly, we focused on these white matter fiber structural features and comprehensively examined the social and biological factors associated with them, such as childhood abuse experiences, current depressive symptoms, parenting practices, and empathy, to identify the risk factors for the development of a vulnerable neurobiological basis in maltreating mothers.

## Results

### Whole-brain tract-based spatial statistics (TBSS)

**Figure 1:**
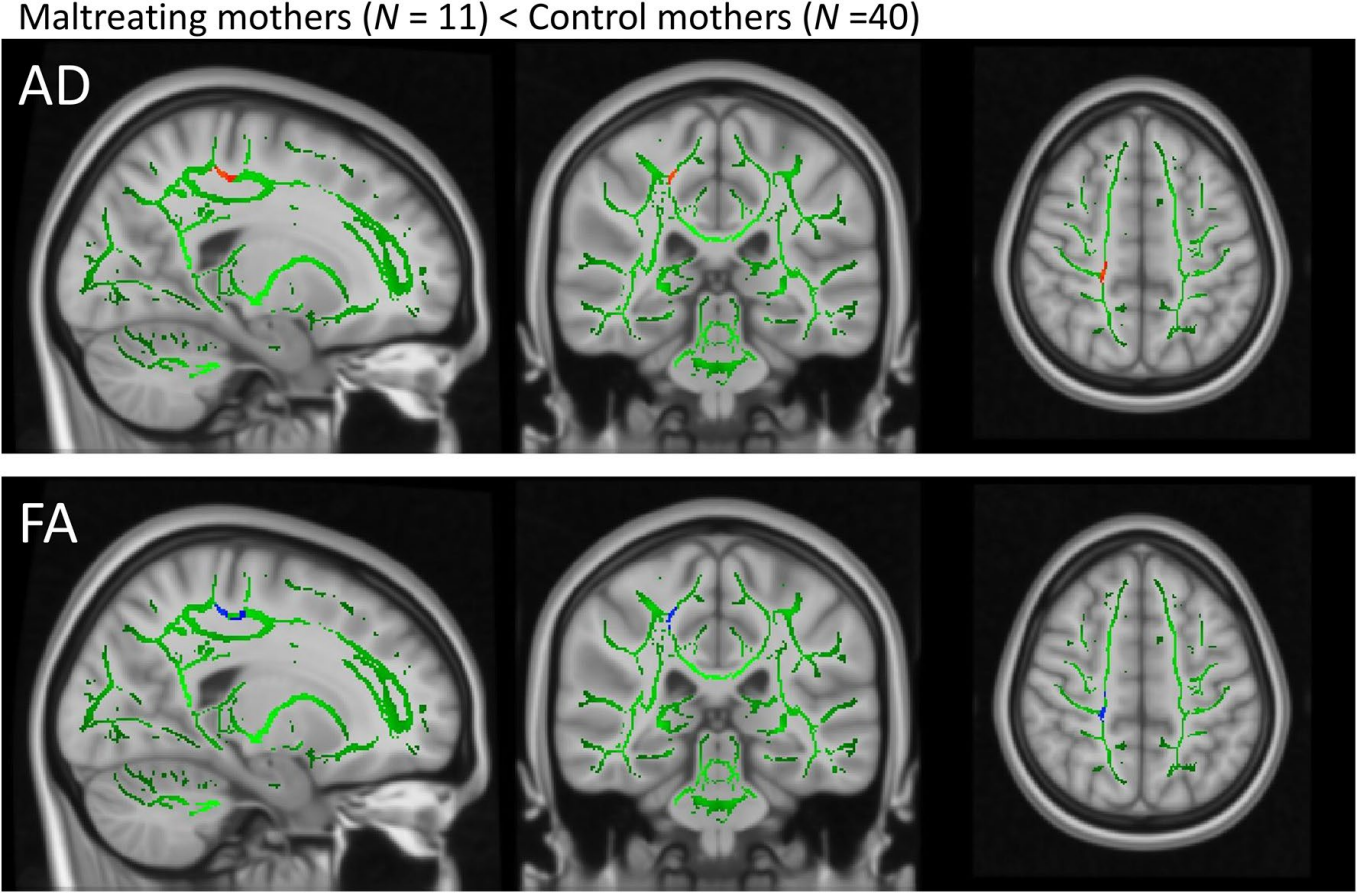
A voxelwise whole-brain TBSS. The right CST showing reduced AD (red, *P* < 0.05, FWE-corrected) and a trend toward reduced FA (blue,* P* < 0.10, FWE-corrected) in the maltreating mothers compared to control mothers. TBSS, tract-based spatial statistics; CST, cortico-spinal tract; AD, axial diffusivity; FWE, family wise error; FA, fractional anisotropy.

There was a significantly reduced AD value (*P* < 0.05, Family Wise Error (FWE)-corrected) and a similar trend in the FA value (*P* < 0.10, FWE-corrected) in the right corticospinal tract (CST) in maltreating mothers compared to control mothers (Table 1 and Fig. 1). There were no significant reductions in mean diffusivity (MD) or RD between maltreating and control mothers. There were no regions with significantly higher values for any image in maltreating mothers than in control mothers.

**Table 1:** Montreal Neurological Institute atlas (MNI) coordinates for statistically significant clusters.

Images	Comparison	TFCE FWE	Cluster	MNI atlas coordinates			Voxel size	Tracts within clusters
				x	y	z		
AD								
	Maltreating < Control	< 0.05	1	20	− 32	49	151	Corticospinal tract
FA								
	Maltreating < Control	< 0.10	1	20	− 32	49	162	Corticospinal tract
	Maltreating < Control	< 0.10	2	23	− 26	40	18	Corticospinal tract

TFCE, threshold-free cluster enhancement; FWE, family wise error; AD, axial diffusivity; FA, fractional anisotropy.

### Region of interest (ROI) correlation analyses with childhood abuse experiences

**Figure 2:**
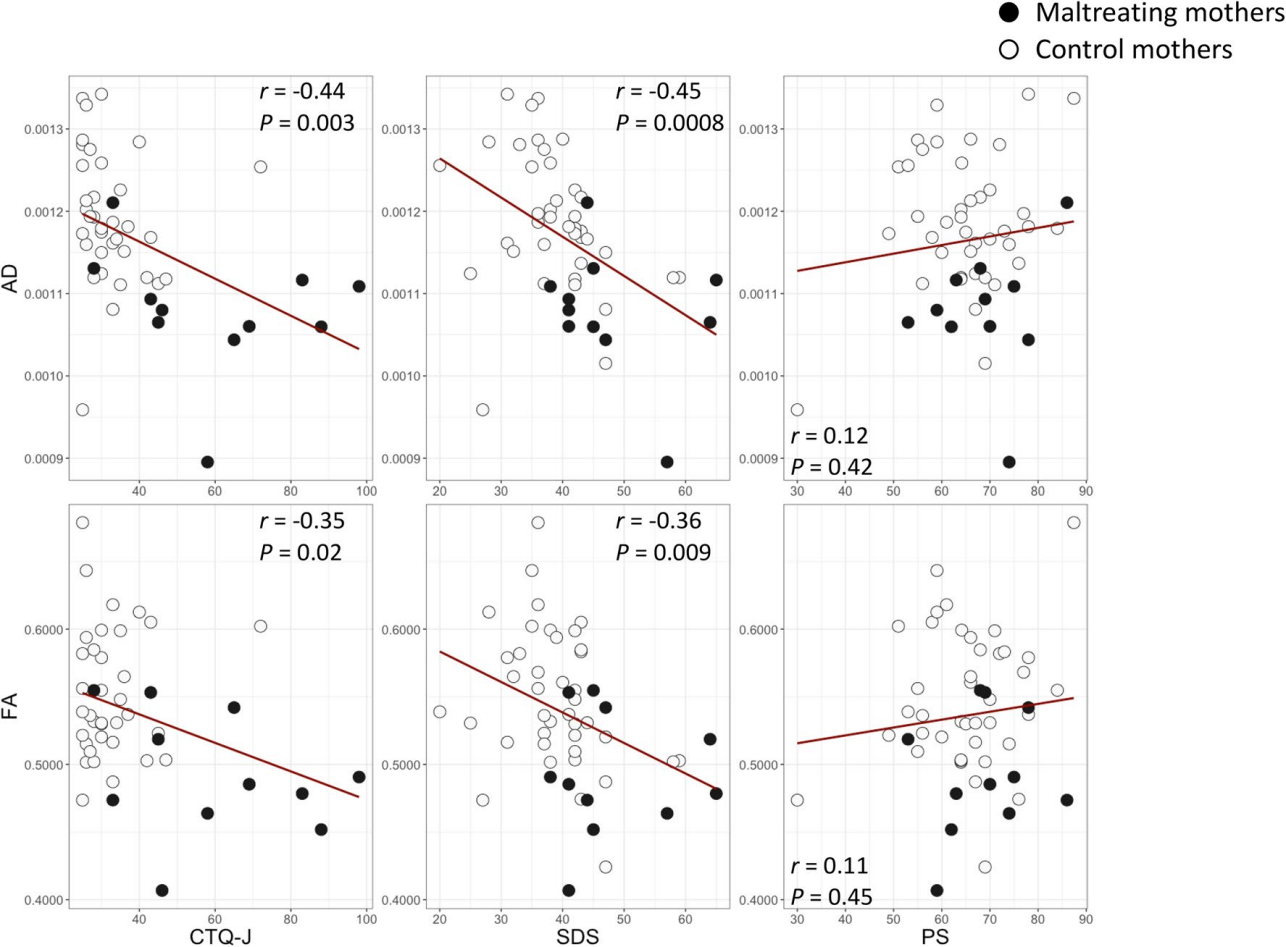
Correlation analyses between white-matter fiber structures and childhood abuse experiences and current psychological states and traits. The scatter plots and regression lines (red) show the association between each questionnaire score and AD and FA in the right CST. AD, axial diffusivity; FA, fractional anisotropy; CST, cortico-spinal tract.

Childhood abuse experiences were assessed using the Japanese version of the Childhood Trauma Questionnaire-Short Form (CTQ-J) (Table 2 and Supplementary Table S1). Across all participants, higher total CTQ-J scores were associated with reduced AD and FA values in the right CST (AD: *r* = − 0.44, *P* = 0.003, Power (1 − β) = 0.91; FA: *r* = − 0.35, *P* = 0.02, Power (1 − β) = 0.72) (Fig. 2). Among the CTQ-J subscales, higher scores for sexual abuse (*r* =− 0.55, *P* = 7.6E−05, Power (1 − β) = 0.99), emotional neglect (*r* = − 0.44, *P* = 0.0027, Power (1 − β) = 0.91), and emotional abuse (*r* = − 0.39, *P* = 0.007, Power (1 − β) = 0.82) were significantly associated with reduced AD (Supplementary Figure S1). Similarly, higher scores for sexual abuse (*r* = − 0.36, *P* = 0.01, Power (1 − β) = 0.75), emotional abuse (*r* = − 0.30, *P* = 0.045, Power (1− β) = 0.58), physical neglect (*r* = − 0.32, *P* = 0.03, Power (1 − β) = 0.64), and physical abuse (*r* = − 0.34, *P* = 0.02, Power (1 − β) = 0.69) were significantly associated with reduced FA values (Supplementary Figure S1).

**Table 2:** The results of the psychological questionnaires.

	Maltreating mother (*N* = 11)	Control mother (*N* = 40)	Statistics*	
CTQ-J				
PA	9 (4.9)	5.3 (0.9)	*t* = − 2.4	*p* = 0.04
EA	14.6 (7.6)	6.4 (2.7)	*t* = − 3.4	*p* = 0.006
SA	7.5 (3.8)	5.2 (0.7)	*t* = − 1.9	*p* = 0.09
PN	12.2 (5.3)	6.8 (2.5)	*t* = − 3.1	*p* = 0.01
EN	16.4 (5.6)	8.8 (3.8)	*t* = − 4.0	*p* = 0.002
Total	59.6 (22.0)	32.5 (9.1)	*t* = − 3.8	*p* = 0.003
SDS	48 (9.1)	38.9 (7.5)	*t* = − 2.9	*p* = 0.01
PS				
OR	40.5 (8.5)	40.2 (9.4)	*t* = − 0.1	*p* = 0.93
LX	28.4 (4.8)	24.9 (4.6)	*t* = − 2.0	*p* = 0.06
IRI-J				
EC	17.8 (2.3)	18.6 (2.6)	*t* = 0.9	*p* = 0.38
PT	16.9 (3.4)	17.3 (3.4)	*t* = 0.3	*p* = 0.74
FS	15.2 (3.2)	13.0 (3.6)	*t* = − 1.9	*p* = 0.08
PD	17.3 (4.7)	14.2 (4.3)	*t* = − 1.9	*p* = 0.08

Statistics presented: Mean (SD), CTQ-J: Childhood Trauma Questionnaire-Short Form, (Bernstein D., 1998, Mizuki&Fujiwara.,2021) PA: Physical abuse, PN: Physical neglect, EA: Emotional abuse, EN: Emotional neglect, SA Sexual abuse, PS: Parenting Scale(Arnold et al.,1993,Itani.2010) OR: Overreactivity, LX: Laxness, IRI: Interpersonal Reactivity Index, (Davis,1980,Himichi et al., 2017) EC: Empathic concern, PT: Perspective taking, FS: Fantasy Scale, PD: Personal distress, SDS: Self-rating Depression Scale (Zung.1965, Fukuda&Kobayashi, 1973).

### ROI correlation analyses with current psychological states and traits

Depressive symptoms, parenting practices, and empathy were assessed using the self-rated depression scale (SDS), Parenting Scale (PS), and Interpersonal Reactivity Index (IRI). Across all participants, higher SDS scores were associated with lower AD and FA values in the right CST (AD: *r* = − 0.45, *P* = 0.0008, Power (1 − β) = 0.92; FA: *r* = − 0.36, *P* = 0.009, Power (1 − β) = 0.75) (Fig. 2). No significant associations were found between the white-matter fiber structure and PS or IRI subscale scores (Fig. 2 and Supplementary Figure S1).

## Discussion

This study was conducted to explore the possibility that white-matter fiber structures are different in abusive mothers compared to control mothers. The results from the case–control analysis showed significantly decreased AD value and a trend toward significantly decreased FA value in the right CST of maltreating mothers. These decreased values in the right CST were associated with childhood abuse experiences and current depressive symptoms in the whole participants of the present study. No association was found between reduced AD and FA values, parenting practices, or empathy. However, it is crucial to understand how these features found in this study are linked to abuse perpetration.

Even though some psychiatric diseases, including postpartum depression and BPD, have been shown by many studies to be risk factors for child abuse and neglect, not all such individuals are abusive. However, since no brain imaging studies have been conducted directly on the perpetrating mothers as in the present study, previous studies have been conducted on those patients, such as postpartum depressed mothers, as a high-risk group, and differences in their white matter fiber structure have been reported. Long et al.reported higher FA and AD values in the right anterior thalamic radiation tract and high FA and low RD values in the cingulum tract in postpartum depression^27^. Silver et al.reported low FA values in the left anterior limb of the internal capsule in depressed mothers 2–8 weeks postpartum^28^. However, no previous attempts have been made to explore the structural characteristics of white matter fiber in maltreating mothers using diffusion weighted images, and our study is the first to identify reduced AD value in the CST and a trend toward reduced FA value in the same nerve bundle. The AD value refers to the magnitude of diffusion parallel to the fiber tract. A lower AD value may reflect damage to the axon, a reduction in axon caliber, and a less coherent orientation of the axon. The FA value refers to the fraction of diffusion that is direction-dependent (anisotropic), and a lower FA value may reflect damage to the myelin sheath surrounding the axon, an enlargement in axon diameter, a reduction in axonal packing density, and an increase in membrane permeability. The MD has a directionally averaged magnitude of diffusion and reflects white matter integrity due to axonal or myelin degradation. RD refers to the magnitude of diffusion perpendicular to the fiber tracts. RD may be relatively more sensitive to myelin and may reflect myelin, axons, and axonal packing density. No differences were observed in the MD and RD values in this study, indicating that FA is not myelin-specific, and that AD is not affected by myelin, making it unlikely that the myelin sheath was abnormal. Reduced AD and FA values may indicate abnormalities in microstructural axonal damage and coherent axonal orientation.

The CST is a typical motor system conduction pathway that descends from the motor cortex to the spinal cord, and is responsible for voluntary movements. A growing consensus suggests that maltreating mothers or mothers at high risk may have exaggerated peripheral nervous system physiological responses to child-related audiovisual stimuli compared to control mothers. Considering exaggerated responses, it was speculated that maltreating mothers may have excessive control over the forcefulness of their voluntary movements when placed in a difficult child-related context. This assumption was demonstrated in an experiment in which maltreating mothers and control mothers were measured and compared in terms of the force with which they held the handgrip apparatus^29^. Therefore, maltreating mothers seem to be vulnerable to the control of force in actual voluntary movements, in addition to the weakness of autonomic nervous system responses. In addition to these physiological and behavioral studies, there are diffusion brain imaging studies that, while not directly examining abusive perpetrators, provide insights into the implications of the low AD and FA values of the CST found in this study. Among psychotic patients, those with a history of violence have been reported to have lower CST integrity than those without^30^. It has also been reported that lower CST integrity is also associated with antisocial behaviors such as aggression and violence^31^, as well as higher impulsivity^32^. The triggering of such aggressive and impulsive behaviors is preceded by the generation of emotion in the limbic system. Emotion regulates cognitive functions and autonomic nervous system responses, but it also affects motor movements via the CST^33^. Given that abusive mothers similarly share a tendency to have higher levels of aggressiveness and impulsivity^34^, the decreased AD value and trend toward lower FA value in the CST revealed in this study may have been the neurobiological findings supporting this manifestation.

The influence of the clinical typology of abuse on the white-matter fiber structure was also examined exploratory, which was classified into physical, emotional, sexual, and neglect, but no significant associations were found. Nine of the eleven maltreating mothers (81.8%) simultaneously committed both physical and emotional abuse. Two mothers committed only neglect (18.1%), and none committed sexual abuse (0%). Therefore, a comparison between the clinical typology of maltreating mothers in some groups was biased toward physical and emotional abuse in the present population and unsuitable for examining the effects. Given this bias, a typological association analysis would require a larger sample size. Considering that most of the maltreating mothers in this study population were active form abusers, in contrast to passive forms representing neglect, it is possible that the observed differences of the CST, which underlies vulnerability of force control of voluntary muscles, may have led to active action, such as excessive violent behaviors to the extent that bruises were formed, strap marks were left, and violent verbal attacks.

This study also examined whether CST features were associated with mothers' childhood abuse experiences. The results showed that reduced AD and FA values in the CST identified from group comparisons were associated with higher CTQ-J scores in the whole participants of the present study. This result tends to be regarded as similar to that of the group comparison, as maltreating mothers are more likely to have experienced childhood abuse than mothers in general (Table 2). However, the results included the following cases: some control mothers had a higher CTQ, and some maltreating mothers had a lower CTQ. Therefore, the findings of significant associations may be regarded as affirming not only the possibility that this reduction in AD and FA values in the CST may have occurred sporadically but also the influence of an intergenerational chain of childhood abuse. Since developmental changes in the white matter of the brain, such as myelination and dendritic pruning, are active during childhood, maltreatment experiences during this period can result in atypical development^35^. Indeed, a study that found a significant association between PTSD and lower FA in the CST^36^ has also been reported, suggesting that traumatic experiences may bring on these features. As more relevant findings, in a series of studies including diffusion brain MRI examining the effects of their childhood abuse experiences on voluntary muscle control in men who have just become fathers, the previously stated handgrip behavioral experiments under the exposures to a context of infant cues showed that excessive force is observed in fathers with a history of childhood abuse experiences^37,38^. These studies also found an association between childhood abuse experiences and the integrity of the uncinate fasciculus (UF) tract, a white matter fiber connecting the amygdala to the prefrontal cortex. However, this study differs in its implications from the present study in that it did not shed light on white matter fiber structures associated with perpetrators of abuse but rather on past traumatic experiences of childhood abuse. Therefore, the fibers found differ from those in the present study. Still, regarding force on voluntary muscle control, they are consistent with the results of the perpetrator mothers in this research group. Our previous study showed that atypical white matter microstructures in the internal capsule of the frontal limbic striatal circuit are involved in emotion regulation in children and adolescents with reactive attachment disorders who have experienced abuse^39^. Therefore, it is suggested that the difference in CST, which is different in site from other similar studies but was also shown to be associated with the CTQ in the present study, may also be one of the findings resulting from developmental changes triggered by childhood maltreatment experiences. If this is the case, caregivers with childhood trauma may have a long-term vulnerability to voluntary muscle control of CST. We plan to examine whether they would perpetrate abuse in the presence of CST vulnerability with a larger sample size.

This study also examined the association between AD and FA values in the CST, depressive symptoms using the SDS, parenting practices using the PS, and empathy using the IRI. The results showed an association between higher depressive symptoms and lower AD and FA values but no association with other indicators. If the vulnerability of the CST was involved in the dominance of voluntary muscles to the extent that they were uncontrollable, the negative influence of the emotional control system on high depressive symptoms was also involved. Neither parenting practices nor empathy were associated with abnormalities in this pathway. This result may be because the overall scores on these scales did not differ between the groups in the first place and did not capture the characteristics of the maltreating mothers.

This study has five main limitations. First, the sample size of the maltreating mothers was small. However, this is a remarkable number of cases because it is the first time that diffusion tensor imaging (DTI) group comparisons have been conducted on mothers who perpetrated abuse. These participants have largely been unaddressed in previous brain imaging studies. Second, we did not evaluate participants' current trauma symptoms. It was noted earlier that emotions affect CST responses. We should have examined the relevance of current trauma symptoms to determine whether trauma symptoms resulting from childhood adversities are related to this structural feature. Third, the structural differences in the CST were found in cross-sectional studies and do not prove a causal relationship, such as that abusive behaviors occur because of them. Fourth, the maltreating mothers who participated in this study were parents who visited our clinic for child outpatient visits and were not clinically evaluated in detail. Structured interviews and common assessments should have been conducted together, not only for symptoms of trauma but also for mental disorders, intellectual disabilities, and personality disorders. A psychiatric examination would have revealed this background and the effects of comorbidities and other illnesses on the brain should have been investigated. Lastly, all maltreating mothers’ children were diagnosed with either ASD or ADHD. Although widely recognized in recent years, there is a clinical concept of developmental trauma disorder (DTD) as defined by Van der Kolk^40^. Therefore, the diagnosis of ASD or ADHD in children may be based on symptoms derived from DTD rather than conventional symptoms of developmental disorders. While it has been noted that children with ASD and ADHD often have emotional and behavioral difficulties that cause psychological distress in their mothers^41^, it is expected that children with DTDs will also have similar symptoms. These psychological loads may be risk factors for maltreatment; however, these confounding effects were not eliminated.

In conclusion, maltreating mothers had vulnerable CST white matter fiber structures. This vulnerability may result from sporadic occurrences or the effects of an intergenerational chain of childhood abuse. Voluntary muscle control via the CST is susceptible to emotion elicitation, and vulnerability to emotion regulation in maltreating mothers may be related to abuse perpetration via vulnerable force control of voluntary muscles. Hence, it may be necessary to consider trauma treatment and neurofeedback therapy for appropriate control of the voluntary muscles in the CST.

## Methods

### Statement

The study protocol was approved by the Ethics Committee of the University of Fukui, Japan (approval nos. 20150068, 20160120, 20220034, and 20220039). This study was conducted in accordance with the Declaration of Helsinki and the Ethical Guidelines for Clinical Studies of the Ministry of Health, Labour, and Welfare of Japan. All the participants provided written informed consent.

### Participants

Based on the concept of RDoC, 11 mothers (mean age ± standard deviation [SD]: 41.9 ± 6.7 years) were enrolled as maltreating mothers in this study by pediatric physicians and child psychiatrists considering they fulfill a research domain criterion defined as currently intervened or having a history of social interventions due to their child abuse conduct (Table 3). All maltreating mothers had conducted physical, emotional, and/or neglect prior to coming into care for their child by the Child Protection Service (CPS). After the CPS intervention, two children lived in a stable environment in local child welfare facilities, and one child lived with a his/her parent who was not a perpetrator after receiving a temporary shelter for CPS. Two were temporarily sheltered and then again lived with their abusive parents with follow-up observations. Six remained in their home staying without ever going through a temporary shelter, but with follow-up observation. All the children were diagnosed with neurodevelopmental disorders according to the DSM-5 criteria (two with attention deficit hyperactivity disorder (ADHD), one with autism spectrum disorder (ASD), and eight with ASD and ADHD comorbidity). Eight maltreating mothers (72.7%) were on mental illness-related medications naïve, except for three (27.3%). Three were depressed, two had obsessive–compulsive disorder (OCD) (including comorbidities), two had ADHD, and one had ADHD and ASD; all three treated with drugs were attending a different clinic other than the children's clinic, and it was unclear to what extent their psychiatric disorders had been diagnosed. No medication washout was requested to participate in the study because of its disadvantages. The control group comprised age-matched 40 mothers (mean age ± SD: 41.1 ± 4.4 years), who were participated in our longitudinal cohort study for child development in the general population. 37 were right-handed in the control mothers. In the maltreating mothers, 10 were right-handed. All the participants were informed about the general aim of the present study (to participate in studying about the formation of mother–child attachment or relationships and their brain structures and functions, avoiding the use of the terms abuse and neglect). The exclusion criteria for all participants were as follows: history of head trauma with loss of consciousness, or perinatal or neonatal complications.

**Table 3:** Demographics of the participants.

	Maltreating mother (N = 11)^a^	Control mother (N = 40)^b^	Statistics*	
Age (year)	41.9 (6.7)	41.1 (4.4)	*t* = − 0.48	*p* = 0.63
Number of children	2.5 (1.1)	2.4 (0.6)	*t* = − 0.35	*p* = 0.73
BMI	25.1 (6.8)	21.6 (3.4)	*t* = − 0.16	*p* = 0.14
Type of abuse				
Physical abuse	9 (81.8)	NA		
Emotional abuse	9 (81.8)	NA		
Sexual abuse	0 (0)	NA		
Neglect	5 (45.5)	NA		
Number of types	2.1 (0.67)	NA		
Cohabitation				
Single	5 (45.5)^c^	2 (5.1)	χ^2^(1) = 11.6	*p* = 0.0007
Spause/partner	6 (54.5)	37 (94.9)^d^		
Level of education				
Middle school or high school graduate	9 (81.8)	7 (18.4)	χ^2^(1) = 15.6	*p* = 0.0008
More than junior college graduate	2 (18.2)	31 (81.6)^e^		
Annual household income (JPY)^f^				
< 3,000,000	2 (28.6)	2 (5.6)	χ^2^(1) = 3.7	*p* = 0.055
> = 3,000,000	5 (71.4)	34 (94.4)		
Comorbid psychiatric disorders	4 (27.3)^g^	1 (2.5)^h^	*t* = − 2.2	*p* = 0.05
History of psychiatric disorders	7 (63.6)	2 (5.0)	*t* = − 3.76	*p* = 0.003
Neurodevelopmental disorders			*t* = − 1.94	*p* = 0.08
ADHD	2 (18.2)	0 (0)		
ASD + ADHD	1 (9.1)	0 (0)		

Statistics presented: Mean (SD); N (%), NA: Not applicable. ^a^Household income; N = 7 ^b^Cohabitation; N = 39, Level of education; N = 38, Household income; N = 36 Four maltreating mothers and four control mothers had no data on household income.

^c^Two maltreating mothers does not cohabit with their children. Those children are under the institutional care. ^d^Thirteen control mothers cohabit with their parent. ^e^Twelve control mothers graduated university or more. ^f^Anunual income of less than 3million yen is recognized as relative poverty. JPY; Japanese yen. 100 JPY is equivalent to $0.7 (June, 2023). ^g^Three maltreating mothers suffer from depression, two mothers suffer from obsessive compulsive disorder, and one mother suffers from panic disorder. ^h^One control mother suffers from depression.

### Psychological measures

#### CTQ-J

Childhood abuse experiences were assessed using the Japanese version of the CTQ-J^42,43^, which is a 28-item scale that uses a 5-point Likert scale with five subscales (emotional abuse, physical abuse, sexual abuse, emotional neglect, and physical neglect). Because four levels of categorical cutoff values were reported for each subscale, the categorical distribution (none, low, moderate, and severe) of the participants in this study is summarized in Supplementary Table S1. The total score was summarized into seven categories, with the addition of intermediate categories between the four categories (Supplementary Table S1).

#### SDS

To determine whether the current depressive symptoms are higher in maltreating mothers and are associated with the structural features of white-matter fibers observed in maltreating mothers, the SDS^44,45^, a widely used depression screening tool, was used. The SDS is a 20-item scale that uses a 4-point Likert scale. The cutoff score was 40, wherein a score of 40 or less indicated "no depression," a score in the 40 s indicated "mild depression," and a score of 50 or more indicated "moderate depression.”

#### PS

To determine whether current parenting practices differ in maltreating mothers and are associated with the structural features of white-matter fibers observed in maltreating mothers, the PS was used, which was developed to measure how parents handle various parenting situations and dysfunctional parenting practices associated with their children's externalizing behaviors^46,47^. The PS is a 30-item scale that uses a 7-point Likert scale with two subscales (overreactivity and laxness). The overreactivity score reflected emotional and harsh discipline, whereas the laxness score reflected permissiveness and inconsistent discipline.

#### IRI

The IRI was used to determine whether the current empathetic trait differed in maltreating mothers and was associated with the white matter fiber structural features observed in maltreating mothers. The IRI (Japanese version) is a 28-item scale that uses a 5-point Likert scale with four subscales (empathic concern [EC], perspective taking [PT], personal distress [PD], and fantasy scale [FS]). EC measures the tendency to evoke other-oriented emotions such as sympathy; PT measures the degree to consider the feelings of others from their point of view; PD measures the degree to which one is preoccupied with the anxiety and fear generated by the observation of others' suffering; and FS measures the tendency to imagine oneself as a character in a story or other fictional character^48^. EC and PD reflect affective aspects, while PT and FS reflect cognitive aspects, with EC and PT being the central concepts of each aspect^49^.

### Diffusion tensor imaging (DTI) data acquisition

Diffusion-weighted images (DWIs) of the 51 participants were acquired using a 3.0 Tesla General Electric SIGNA MRI system (Signa PET/MR, GE Healthcare, Milwaukee, WI, USA) with an 8-channel head coil. DWIs were obtained using a spin-echo echo planar imaging (EPI) sequence (repetition time [TR] = 9352 ms, echo time [TE] = 74.6 ms, flip angle = 90°, field of view [FOV] = 100 × 100 mm, matrix size = 256 × 256, slice thickness = 3 mm with no gap, 1395 slices). Moreover, diffusion MRI using a single phase-encoding (PE) direction, most commonly anterior–posterior (AP) direction was acquired, using a spin-echo EPI sequence (TR = 9352 ms, TE = 74.6 ms, FOV = 100 × 100 mm, slice thickness = 3 mm with no gap, 256 slices). The DWIs were isotropically distributed along 30 directions (*b* value = 1,000 s/mm^2^) and one non-diffusion-weighted image (*b* value = 0 s/mm^2^; b = 0 images). All the participants were instructed to remain motionless in the scanner. The head movements were minimized by stabilizing the head with a cushion.

### DTI pre-processing

DTI data, originally in the Digital Imaging and Communication in Medicine image format, were converted to the Neuroimaging Informatics Technology Initiative image format using the MRIcron software (https://www.nitrc.org/projects/mricron). The DTI data were pre-processed using MRtrix 3.0.4 (https://mrtrix.org)^50^ and the Tract-based spatial statistics (TBSS) pipeline at the Oxford Centre for Functional MRI of the Brain Software Library (FSL; https://www.fsl.fmrib.ox.ac.uk)^51^. All posterior-anterior (PA) DWIs were denoised^52^, and Gibbs ringing artifacts were removed^53^. The b = 0 images of the PA (*b* = 0 s/mm^2^) and anterior–posterior (AP) (*b* = 0 s/mm^2^) PE scans were used as references in the subsequent FSL steps^54^. Susceptibility-induced off-resonance field and eddy current and movement-induced distortions were corrected using the Topup tool^54,55^ and eddy tool^56^, respectively. Bias field correction was performed using the Advanced Normalization Tools N4 bias correction algorithm^57^. The masks were generated using the bias-field-corrected images. The FSL toolbox DTIFIT fits the pre-processed image based on four parameters of the diffusion tensor model to yield the FA, mean diffusivity (MD), and AD, which were quantified by eigenvalues (L1, L2, and L3). L1 corresponded to AD, whereas the average values of L2 and L3 corresponded to RD^58^.

### TBSS

We used TBSS, a voxel-wise approach, to examine whole brain fiber tracts for better sensitivity, objectivity, and interpretability of DTI analyses, as previously described^39^. The TBSS tool in FSL was used for the voxel-wise analysis of the preprocessed FA, MD, AD, and RD values. Individual FA, MD, AD, and RD images were aligned to the FMRIB58_FA_1 mm template and transformed into the Montreal Neurological Institute standard space. Next, all the aligned FA images were averaged to generate a mean FA image, which was then thinned to create a mean tract skeleton. The tract skeleton was thresholded at an FA value of ≥ 0.2 to exclude peripheral tracts and minimize the partial volume effect. Finally, each participant's aligned FA, MD, AD, and RD images were projected onto the mean tract skeleton and the resulting data were fed into a voxel-wise permutation-based analysis.

### Statistical analysis

Demographics and clinical characteristics were compared using chi-squared (χ^2^) tests, and *t*-tests, using R^59^. The voxel-wise statistical analysis of the FA, MD, AD, and RD data was performed using ‘randomize,’ a non-parametric permutation-based inference approach, and a standard general linear model (GLM) design matrix applied in FSL, using the threshold-free cluster enhancement option^60^. To compare the differences in the FA, MD, AD, and RD values between the two groups and controls for confounding variables, age was entered into the GLM as a covariate of no interest. The number of permutations was set to 5,000, and multiple voxel-wise comparisons were controlled using FWE correction with a threshold set to *P* < 0.05 and a cluster size of ≥ 10 voxels separately across the four different outcome measures. The anatomical locations of significant clusters were identified using the JHU ICBM-DTI-81 white matter labels implemented in the FSL.

### Supplementary Information

Supplementary Information.

## Data Availability

The datasets generated during and/or analyzed during the current study are not publicly available due to the risk of subject identification, subjects whose anonymity should be carefully protected. However, the data are available from the corresponding authors on reasonable requests.
