# Supplementary material for: Diffusion tensor imaging of white-matter structural features of maltreating mothers and their associations with intergenerational chain of childhood abuse
Source: Sci Rep. 2024 Mar 7;14:5671. doi: 10.1038/s41598-024-53666-0 (PMC10920819; doi:10.1038/s41598-024-53666-0)
Supplement: Supplementary file 1 — Supplementary Information. [file 41598_2024_53666_MOESM1_ESM.pdf]

Supplementary Table S1. Categorical distribution of the participants for their childhood abuse experiences.

	Maltreating mothers	Control mothers
n	11	35[a]
EA		
None (5-8)	3 (0.27)	31 (0.89)
Low (9-12)	1 (0.09)	3 (0.09)
Moderate (13-15)	2 (0.18)	0 (0)
Severe (>16)	5 (0.45)	1 (0.03)
PA		
None (5-7)	6 (0.55)	34 (0.97)
Low (8-9)	1 (0.09)	0 (0)
Moderate (10-12)	2 (0.18)	1 (0.03)
Severe (>13)	2 (0.18)	0 (0)
SA		
None (5)	6 (0.55)	32 (0.91)
Low (6-7)	1 (0.09)	1 (0.03)
Moderate (8-12)	3 (0.27)	2 (0.06)
Severe (>13)	1 (0.09)	0 (0)
EN		
None (5-9)	2 (0.18)	23 (0.66)
Low (10-14)	3 (0.27)	8 (0.23)
Moderate (15-17)	1 (0.09)	3 (0.09)
Severe (>18)	5 (0.45)	1 (0.03)
PN		
None (5-7)	2 (0.18)	23 (0.66)
Low (8-9)	3 (0.27)	8 (0.23)
Moderate (10-12)	3 (0.27)	3 (0.09)
Severe (>13)	3 (0.27)	1 (0.03)
Total		
None (25-31)	1 (0.09)	21 (0.60)
None to Low (32-40)	1 (0.09)	9 (0.26)
Low (41-51)	3 (0.27)	4 (0.11)
Low to Moderate (52-55)	0 (0)	0 (0)
Moderate (56-68)	2 (0.18)	0 (0)
Moderate to Severe (69-72)	1 (0.09)	1 (0.03)
Severe (73-125)	3 (0.27)	0 (0)

EA: Emotional abuse, PA: Physical abuse, SA: Sexual abuse, EN: Emotional neglect, PN: Physical neglect. Parenthesis represents rates of the number. [a]Five participants were not examined CTQ-J. According to the defined four levels of categorical cutoff values for each subscale, the categorical distribution (none, low, moderate, and severe) of the participants in this study is summarized. The total score was summarized into seven categories, with the addition of intermediate categories between the four categories.

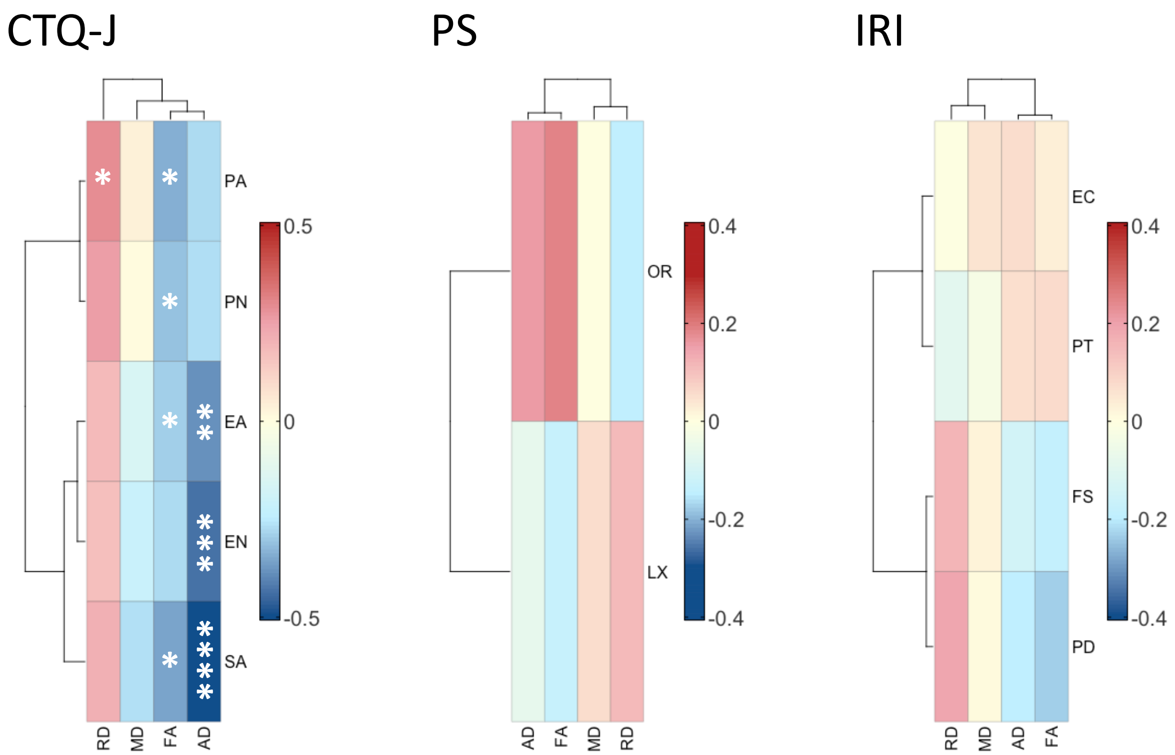

Supplementary Figure S1. Association between white-matter fiber structures of the CST and subscales for CTQ-J, PS, and IRI.
PA: Physical abuse, PN: Physical neglect, EA: Emotional abuse, EN: Emotional neglect, SA Sexual abuse, OR: Overreactivity, LX: Laxness, EC: Empathic concern, PT: Perspective taking, FS: Fantasy Scale, PD: Personal distress.
Color bar represents correlation coefficient (*r*).
Among the CTQ-J subscales, higher scores for sexual abuse (r = -0.55, P = 7.6E-05), emotional neglect (r = -0.44, P = 0.002), and emotional abuse (r = -0.39, P = 0.007) were significantly associated with reduced AD (Supplementary Figure S1). Similarly, higher scores for sexual abuse (r = -0.36, P = 0.01), emotional abuse (r = -0.30, P = 0.045), physical neglect (r = -0.32, P = 0.03), and physical abuse (r = -0.34, P = 0.02) were significantly associated with reduced FA values.
*: $P < 0.05$, **: $P < 0.01$, ***: $P < 0.005$, ****: $P < 0.001$
